# Understanding photoacoustic signal formation in the presence of transparent thin films

**DOI:** 10.1016/j.pacs.2024.100617

**Published:** 2024-05-13

**Authors:** Maksym Illienko, Matthias C. Velsink, Stefan Witte

**Affiliations:** a Advanced Research Center for Nanolithography (ARCNL), Science Park 106, Amsterdam, 1098 XG, The Netherlands; b Department of Physics and Astronomy, Vrije Universiteit, De Boelelaan 1081, Amsterdam, 1081 HV, The Netherlands

**Keywords:** Ultrafast photoacoustics, Strain-optic, Strain waves, Strain detection, Pump-probe spectroscopy

## Abstract

Strain-induced variation of the refractive index is the main mechanism of strain detection in photoacoustic experiments. However, weak strain-optic coupling in many materials limits the application of photoacoustics as an imaging tool. A straightforward deposition of a transparent thin film as a top layer has previously been shown to provide signal enhancement due to elastic boundary effects. In this paper, we study photoacoustic signal formation in metal covered by thin transparent films of different thicknesses and demonstrate that in addition to boundary effects, the photoacoustic response is affected by optical effects caused by the presence of the top layer. The interplay of optical effects leads to a complex temporal signal shape that strongly depends on the thickness of the thin film.

## 1 Introduction

**Fig. 1:**
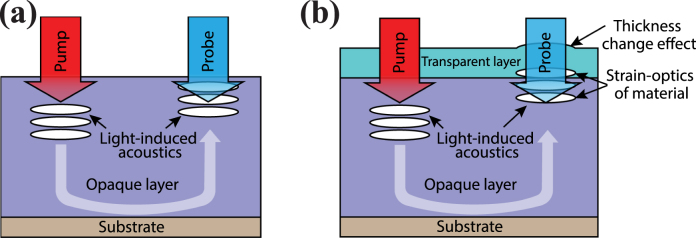
The schematic of the acoustics generation and detection process in a single opaque layer (a) and a sample with a transparent top layer (b).

For the purpose of nanoscale imaging and detection through optically opaque materials, high-frequency ultrasound can become an intermediate carrier of information about buried nanostructures [1], [2], [3], [4]. As sound frequencies on the order of several hundred gigahertz correspond to an acoustic wavelength of only tens of nanometers in most materials, such acoustic waves may be used for high-resolution imaging [3], [4], [5]. Probably the most straightforward way to generate such high frequency acoustic waves is to irradiate the sample of interest with ultrafast laser pulses [6]. Due to rapid local heating caused by the pump laser pulse absorption, the area of the sample near the surface undergoes thermal stress which becomes a source of acoustic (strain) waves. These strain waves propagate longitudinally into the sample and are reflected from any internal interfaces or structures. The resulting acoustic echoes cause refractive index variations near the surface of the sample that can be detected by a time-delayed probe laser pulse (Fig. 1(a)). The imaging or detection of internal structures of the sample of interest can be done by investigating the probe reflectivity change $\Delta R/R_{0}$ dependance on the pump-probe delay [1], [2], [3].

A weak strain-optic coupling in many materials and low strain amplitudes of laser-induced strain pulses lead to a very low reflectivity change obtained in experiments. To achieve a satisfactory signal-to-noise ratio, the measurement time needs to be long, limiting the application of photoacoustics in industry. Improving signal levels by increasing pump pulse energy is limited by the finite optical damage threshold of materials. In general, strategies to increase signal ideally result in a stronger coupling between the strain wave and the probe light. Therefore, a detailed understanding of such photoelastic interactions is important, and provides a route towards signal optimization and the design of improved photoacoustic transducers. Furthermore, in many applications the strain generation does not take place at the surface of a structure, but at the interface between an opaque material and a transparent coating layer. In such cases, the generated strain may travel into the transparent layer, resulting in additional photoacoustic signal contributions such as Brillouin oscillations and vibrations of transparent layer interfaces [7], [8], [9], [10], [11], [12]. A specific approach to increasing detection sensitivity is to add a partial reflector above the material under study, forming a Fabry–Perot resonator that can be designed to strongly enhance the optical response to strain variations [13]. Here, we study the photoacoustic response of metals covered by transparent thin films with a thickness at or below the spatial extent of the strain pulse. Such stack configurations are found in various applications, ranging from precision optical coatings to photoresists in nanolithography. The use of photoacoustic metrology in such applications requires a detailed understanding of the signal formation in the presence of thin transparent films. In previous experiments, we observed signal enhancements in such a geometry [14]. We now investigate the origin of this enhancement, and identify several contributions to the photoelastic interaction. The interplay between these effects results in a complex temporal response, with signals that differ considerably from the characteristic Brillouin oscillations observed in thicker transparent layers.

In the presence of a transparent layer, the total $\Delta R/R_{0}$ signal is made up of three contributions: a thickness change of the transparent layer, strain-induced refractive index variations in the transparent layer, and strain-induced refractive index variations in the opaque layer (Fig. 1(b)). For small changes of reflectivity, the total $\Delta R/R_{0}$ signal can be treated as a linear sum of these three contributions. An additional effect is that in the presence of a transparent top layer, the $\Delta R/R_{0}$ variation due to strain in the opaque layer itself will be different. We call this difference an optical coating effect of the transparent layer. Depending on material parameters such as strain-optic coupling and nominal refractive index, and the thickness of the transparent layer, the $\Delta R/R_{0}$ signal can be dominated by either of the three mentioned effects, leading to significant variations in signal shape and amplitude.

The deposited transparent top layer acts as a Fabry Perot interferometer (FPI) attached to the sample. When the strain wave returns to the sample surface it causes changes in FPI parameters, affecting the $\Delta R/R_{0}$ signals. An FPI-based detection system can significantly enhance detection sensitivity when designed for that purpose [13]. In our configuration, the strain wave propagates through the entire ‘interferometer’. Furthermore, FPI-type sensors are used for ultrasonic detection [15], [16] and photoacoustic imaging in biomedicine [17]. However, in biomedical applications, the optical pump pulse usually propagates into the sample and is absorbed by internal features of interest. Those features become sources of strain waves which are then detected on the sample surface. Also in that application, the FPI sensors themselves have no effect on the generation process of strain waves. Conversely, in our case, both the generation and detection of strain waves happen near the surface of the sample and any modification of the surface also influences strain generation.

## 2 Theoretical model

To study the process of strain waves generation and detection in bi-layered structures, we use a physical model based on earlier work of our group [18]. Since the pump illumination area is typically much larger than both the total thickness of a sample and generated strain wavelength, we neglect any diffraction effects and only consider a one-dimensional problem.

Physical samples are fabricated by sequential deposition of poly(methyl methacrylate) (PMMA), metal, and transparent layers onto glass substrates (see Section 3). The measured time delay range is significantly smaller than the acoustic roundtrip time in the PMMA layer. Thus, the simulation domain excludes glass substrates considering a three-layer structure with free surfaces.

The model consists of a set of differential equations that we solve numerically using the finite-difference time-domain (FDTD) method [19], [20], [21], [22].

**Fig. 2:**
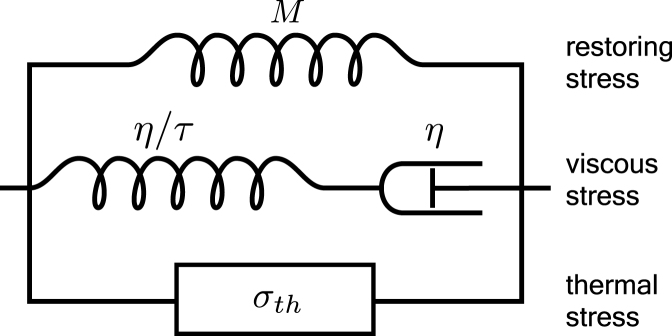
Standard linear solid model. Total stress is splitted into restoring $\sigma_{re}$, viscous $\sigma_{v}$, and thermal $\sigma_{th}$ components. Restoring stress comes from restoring force due to body deformation. Viscous stress emulates friction forces due to rate of strain. Thermal stress originates from the change of lattice temperature.

### 2.1 Generation of strain waves

Strain waves are generated due to the thermoelastic effect after a rapid lattice temperature increase [6], [23]. To calculate lattice temperature evolution we use a hyperbolic two-temperature model [24], [25], [26] described by the following coupled equations

(1) $$C_{e}\left(T_{e}\right)\frac{\partial T_{e}}{\partial t}+\frac{\partial Q_{e}}{\partial z}=-G\left(T_{e}-T_{l}\right)+S\left(z,t\right),$$

(2) $$\tau_{e}\frac{\partial Q_{e}}{\partial t}+Q_{e}=-k_{e}\left(T_{e},T_{l}\right)\frac{\partial T_{e}}{\partial z},$$

(3) $$C_{l}\frac{\partial T_{l}}{\partial t}=G\left(T_{e}-T_{l}\right),$$

where $T_{e}$ and $T_{l}$ are the electron and lattice temperature respectively, $Q_{e}$ is the electron heat flux, $C_{e}$ and $C_{l}$ are the electron and lattice heat capacity, $k_{e}$ is the electron thermal conductivity, $\tau_{e}$ is the electron relaxation time, G is the electron–phonon coupling constant, and S is the source defined by pump light absorbed power density. The temperature dependences of $C_{e}\propto T_{e}$ and $k_{e}\propto \left(T_{e}/T_{l}\right)$ are included [26], [27]. Values for $C_{e}$ and G are taken from [27], and for $k_{e}$ from [28]. The electron relaxation time is theoretically calculated as $\tau_{e}=3k_{e}/v_{F}^{2}C_{e}$ [29], where $v_{F}$ is the Fermi velocity. The temperature dependence of $\tau_{e}$ is neglected.

The two-temperature model is sufficient to describe ultrafast thermodynamics in metals. In dielectrics the heat conductivity is determined by heat transfer via the lattice, which is much weaker than via free electrons in metals [30]. We therefore exclude heat transport effects in the top dielectric and bottom PMMA layers. Also, since the dielectric layers are not absorptive for the pump wavelength used in our experiments, the strain generation is localized within the metal layer. Thus, temperature dynamics are only considered in a metal film with thermal-insulation boundary conditions $Q_{e}=0$ applied to the dielectric-metal and metal-substrate interfaces, and initial conditions:

(4) $$T_{e}\left(z,t=0\right)=T_{l}\left(z,t=0\right)=300\;K,\;Q_{e}\left(z,t=0\right)=0.$$

### 2.2 Propagation of strain waves

To simulate the propagation of strain waves including viscous damping, we use the theory of linear elasticity in combination with a standard linear solid (SLS) model [31], [32]. A graphical representation of the model is shown in Fig. 2. The stress–strain relation equations for the depicted SLS model together with equations of motion form a phenomenological model of a viscoelastic system:

(5) $$\sigma_{re}=Mɛ,$$

(6) $$\sigma_{v}=-\tau \frac{\partial \sigma_{v}}{\partial t}+\eta \frac{\partial ɛ}{\partial t},$$

(7) $$\sigma_{th}=-3K\alpha \Delta T_{l},$$

(8) $$\frac{\partial ɛ}{\partial t}=\frac{\partial v}{\partial z},$$

(9) $$\rho \frac{\partial v}{\partial t}=\frac{\partial }{\partial z}\left(\sigma_{re}+\sigma_{v}+\sigma_{th}\right),$$

where $\sigma_{re}$, $\sigma_{v}$ and $\sigma_{th}$ are restoring, viscous and thermal stress respectively, ɛ is the strain, v is the velocity, ρ is the mass density, M is the P-wave modulus, K is the bulk modulus, α is the thermal expansion coefficient, τ is the phenomenological relaxation time, η is the phenomenological bulk viscosity. The thermal stress $\sigma_{th}$ forms a source of strain waves, which is included in the model as a thermoelastic effect [6]. The initial conditions for Eqs. (5)–(9) imply zero values of stress, strain, and velocity. Edge boundaries are considered to be free corresponding to zero stress at the front surface of the transparent layer and the back surface of the PMMA layer. Interlayer boundary conditions suggest the continuity of stress and velocity across material interfaces. In numerical calculations, interlayer boundaries are satisfied by harmonic and arithmetic averaging of elastic moduli and densities at material discontinuities [21], [22].

### 2.3 Detection of strain waves

The strain waves give rise to a change in reflectivity through the photoelastic effect. For a one-dimensional problem and longitudinal strain waves the change of permittivity is

(10) $$\Delta \epsilon_{ɛ}=-\epsilon^{2}P_{12}ɛ,$$

where ϵ is the nominal permittivity and $P_{12}$ is the component of the complex photoelastic tensor. In our case, it is more convenient to consider changes in a refractive index rather than in a dielectric permittivity. Thus, from Eq. (10) we get

(11) $$\Delta n_{ɛ}=\frac{\partial n}{\partial ɛ}ɛ,$$

where $\partial n/\partial ɛ=-P_{12}n^{3}/2$ is the strain-optic coefficient. From now we will use the term strain-optic effect instead of photoelastic effect, meaning that strain induces changes in a refractive index.

In general, refractive index changes can also result from variations in electron and lattice temperature. The rise of lattice temperature in our experiments is only in the order of tens of degrees Kelvin. Thus, the refractive index change can be approximated by a linear relationship with respect to the lattice temperature increase:

(12) $$\Delta n_{T_{l}}=\frac{\partial n}{\partial T_{l}}\Delta T_{l}.$$

Conversely, in the first several femtoseconds after pump absorption, the electron temperature typically increases by several hundred or even thousand K. Such a significant temperature variation can lead to a non-linear response of the refractive index. This behavior can be very complicated and strongly depends on the material. The time for electrons and lattice to reach thermal equilibrium is typically less than 1 ps [26]. In our simulations, considering the temperature dependence of heat capacity and diffusion, this thermalization time does not exceed 5 ps. Since we are mainly interested in acoustic phenomena at later time delays, we exclude electron thermo-optic effects from our model. The total change of refractive index due to strain and thermo-optic effect is then defined as $\Delta n=\Delta n_{ɛ}+\Delta n_{T_{l}}$.

To solve Eqs. (1)–(3) and (5)–(9) numerically, a stratified thin film medium is considered. Thus, the whole medium is a stack of thin layers of different refractive index. We calculate the reflectivity of such a stack with the transfer matrix method [33]. In addition, the thickness change of each individual layer due to strain is considered.

## 3 Experimental setup and samples

**Fig. 3:**
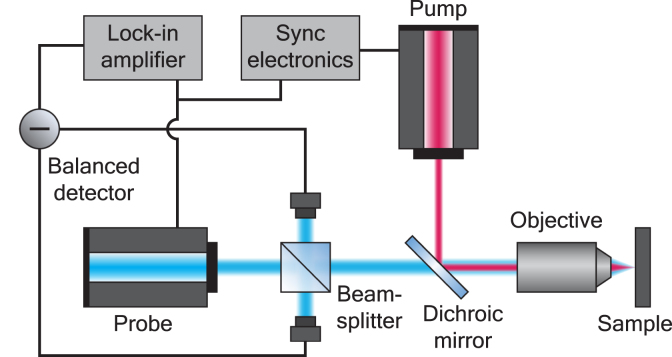
Simplified schematic of the experimental setup used for time-resolved photoacoustic spectroscopy. See main text for details.

Experiments were performed with a setup developed in our group [34], which incorporates the pump-probe technique via modulated asynchronous optical sampling. The schematic of the setup is shown in Fig. 3. The pump source is an Ytterbium-doped fiber laser operating at 1030 nm wavelength, generating pulses with 180 fs duration at 50 MHz repetition rate (Menlo System Orange). The probe source is the frequency-doubled output of an Erbium-doped fiber laser at 780 nm wavelength, which emits 70 fs pulses at 100 MHz repetition rate (Menlo System C-Fiber 780). The probe pulses can be further upconverted to a wavelength of 390 nm through second harmonic generation in a beta-barium borate (BBO) crystal. The pump laser is electronically synchronized to the free-running probe laser, and the pump-probe delay can be tuned by adding a controlled offset to the pump laser repetition frequency [35].

Both pump and probe laser beams are collinearly focused by a microscope objective (Olympus LUCPLFLN20X, x20, N.A. 0.45) onto the sample surface. The reflected probe beam is filtered by a dichroic mirror and detected in one of the ports of a multimode fiber-coupled balanced detector (500 MHz, Femto HBPR), with a reference beam straight from the probe laser in the other port. The detector signal is then amplified by a lock-in amplifier (Zurich Instruments UHFLI) at 50 MHz demodulation frequency.

We use 780 nm and 390 nm probe light to measure strain-induced reflectivity change in aluminum and gold, respectively. Different probe wavelengths are used to maximize strain-optic coupling in a particular material.

The experimental samples were fabricated on glass coverslips (borosilicate 0.5 mm). To reduce losses of strain waves due to coupling into the glass substrate, a layer of 1 µm-thick PMMA layer was first deposited by spin coating. PMMA has a significantly lower acoustic impedance than the metals used in our experiments. Thus, a strain wave that propagates to the rear side of the metal layer is strongly reflected from the metal-PMMA interface. The metal layers (gold and aluminum) and transparent layers of varying thickness (${\mathrm{Al}}_{2}O_{3}$) were deposited by electron beam physical vapor deposition (E-Flex, Polyteknik).

## 4 Results and discussion

### 4.1 Photoacoustic signals in aluminum films

**Fig. 4:**
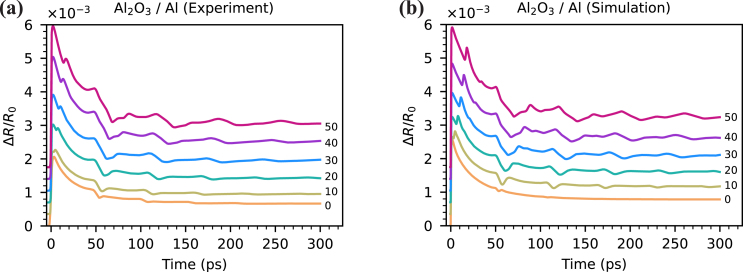
Measured (a) and simulated (b) reflectivity change in ${\mathrm{Al}}_{2}O_{3}$/aluminum samples. Pump pulse energy 1 nJ. Probe wavelength 780 nm. Aluminum thickness 170 nm. ${\mathrm{Al}}_{2}O_{3}$ thickness is shown on the graph in nm. A vertical offset between the different traces is applied for clarity. Optical parameters of aluminum used in simulation: $n_{pump}=1.4+9.85i$, $n_{probe}=2.65+8.46i$, ∂n/∂ɛ=−29.19−25.15i, and $\partial n/\partial T_{l}=\left(-1.18+1.62i\right)\times 10^{-3}\;K^{-1}$. Strain-optic and thermo-optic coefficients are taken from [36]. The magnitude of these coefficients is adjusted for better agreement with experiments keeping the ratio of real and imaginary parts. Optical parameters of ${\mathrm{Al}}_{2}O_{3}$: $n_{pump}=1.67$, $n_{probe}=1.67$, and ∂n/∂ɛ=−0.4. The strain-optic coefficient of ${\mathrm{Al}}_{2}O_{3}$ is adjusted for better agreement with experiments.

Fig. 4(a) shows the reflectivity change due to laser-induced strain waves in a 170 nm thick aluminum film with ${\mathrm{Al}}_{2}O_{3}$ top layer. The first echo reaches the front surface around 50 ps after the pump excitation, causing a dip in the reflectivity curves. This dip repeats approximately every 50 ps corresponding to the roundtrip time in this ${\mathrm{Al}}_{2}O_{3}$/aluminum structure. At zero pump-probe time delay, a peak is observed both in simulations (Fig. 4(b)) and experiments. The origin of this peak is the thermo-optic effect and thermal strain near the surface of the aluminum film. Supplementary Movie 1 shows a simulation of the propagating strain pulse in the aluminum layer, along with the resulting time dependent $\Delta R/R_{0}$ signal.

The experimental curves show strong echo decay caused by sound attenuation in aluminum and strain wave coupling into the PMMA layer. The sound attenuation is frequency dependent and acts as a lowpass filter leading to signal smoothing and dispersive broadening. In simulations, we adjusted the aluminum viscosity parameter $\eta =5\times 10^{-3}\;Pa\;s$ to optimize the shape of echoes. The coupling of strain waves into the PMMA layer is also significant. For speed of sound $\nu_{Al}=6440\;m/s$, $\nu_{PMMA}=2750\;m/s$ and density $\rho_{Al}=2700\;kg/m^{3}$, $\rho_{PMMA}=1190\;kg/m^{3}$ the acoustic impedance mismatch leads to 68 % reflectivity at the aluminum-PMMA interface. The thickness of the deposited PMMA layer is around 1 µm corresponding to a roundtrip time more than 700 ps. Hence, there are no strain waves coupled back into aluminum from the PMMA layer within the measured pump-probe delay range. In simulations P-wave modulus of PMMA is reduced while density is increased, both by factor 5. This results in 5 times lower speed of sound but does not affect acoustic impedance, keeping acoustic reflectivity at the aluminum-PMMA interface the same. Thus, the thickness of the PMMA layer can be reduced to save computational time.

The echo amplitude increases and the shape of the reflectivity curves changes with the thickness of ${\mathrm{Al}}_{2}O_{3}$. Strain waves generated at the surface of the aluminum also propagate into the ${\mathrm{Al}}_{2}O_{3}$ layer. After a round trip in ${\mathrm{Al}}_{2}O_{3}$ the strain wave partially enters the aluminum film causing an additional peak around 10–20 ps time delay. For thicker ${\mathrm{Al}}_{2}O_{3}$ layers the roundtrip time is longer, resulting in an increased time delay of this peak.

**Fig. 5:**
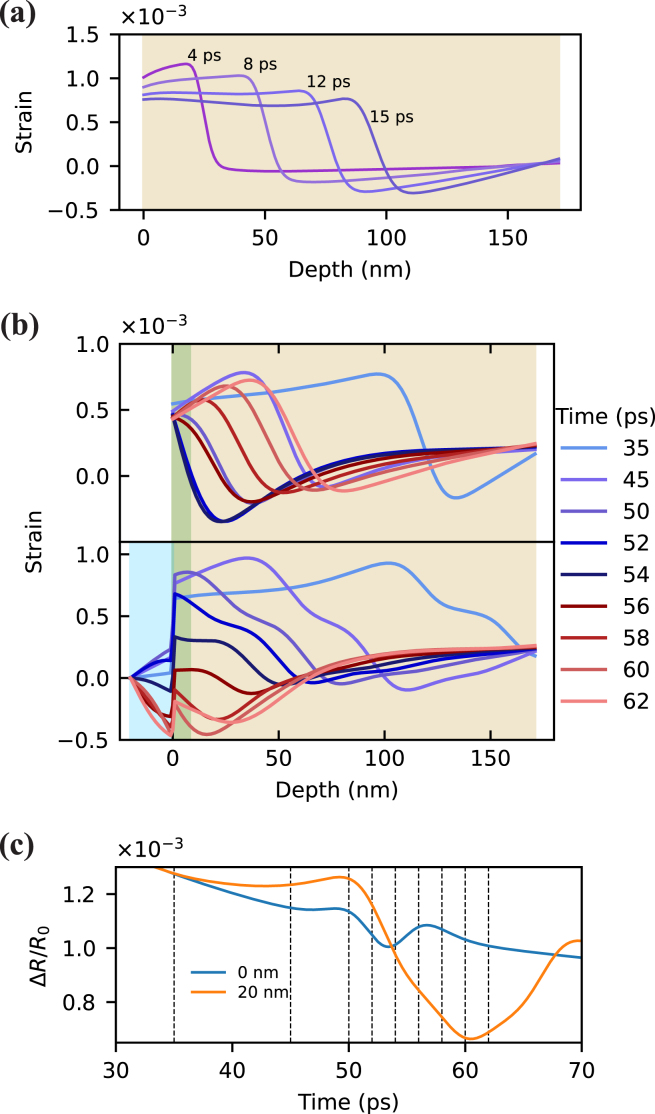
Simulation of light-induced strain waves in the ${\mathrm{Al}}_{2}O_{3}$/aluminum structure. Beige filling depicts aluminum, blue – ${\mathrm{Al}}_{2}O_{3}$, green – penetration depth of 780 nm probe light into aluminum. Penetration depth is defined as λ/4πκ, where κ is the imaginary part of the refractive index n=2.65+8.45i. (a) Strain wave evolution at increasing time delays after pump excitation from the left in a 170 nm thick aluminum film. (b) The strain wave shape at different time delays, around the expected return time at the front surface. Top subplot is a bare aluminum film, bottom — with 20 nm of ${\mathrm{Al}}_{2}O_{3}$. Because of the different elastic constants of aluminum and ${\mathrm{Al}}_{2}O_{3}$, the strain experiences a discontinuity at the interface between the materials. (c) Reflectivity change around the first echo for bare aluminum (blue) and in the presence of a 20 nm ${\mathrm{Al}}_{2}O_{3}$ layer (orange). The vertical dashed lines correspond to the time labels in (b).

The echoes from the aluminum layer are found to increase in signal strength for thicker ${\mathrm{Al}}_{2}O_{3}$ layers. This enhancement is caused by free surface elimination [14] and the optical effects mentioned in Section 1. To understand the free surface effect, consider a strain pulse generated in an aluminum film (Fig. 5(a)). After the pump absorption, two negative counter-propagating unipolar strain pulses are generated. One of them propagates into the sample while another one immediately reflects from the free surface and undergoes a sign flip [6], [8]. This results in a bipolar pulse with a negative part followed by a positive strain. If thermal diffusion and dispersion effects are excluded, the strain pulse has the shape of two mirrored truncated exponential functions with an infinite gradient in the point of symmetry. Thermal diffusion and strain wave dispersion lead to pulse broadening and smoothing, which results in a sine-like shape of the strain pulse. Upon the reflection from a free surface or any interface with lower acoustic impedance material, the pulse undergoes a sign flip. Thus, the strain distribution near the free surface is effectively a sum of two mirrored counter-propagating pulses with opposite phases. Because the strain pulse generated in aluminum has a bipolar shape, the strongest strain field in the vicinity of the surface occurs when the leading half of the pulse is fully reflected. At this moment the reflected part constructively interferes with the following rear half of the pulse. This can be observed in the top subplot of Fig. 5(b) at 54 ps. However, due to the non-instantaneous transition between negative and positive parts of a strain pulse, the point of maximum strain does not reach the surface. Furthermore, strain at the surface is fixed to its thermal value [14]. That is why all the curves in the top subplot of Fig. 5(b) have almost the same value at the edges. The small deviations are caused by thermal decay in aluminum. As optical detection of the strain wave happens in a thin layer defined by the probe penetration depth (indicated by the green area in Fig. 5(b)), this situation results in a small detectable reflectivity change (Fig. 5(c), blue). In contrast, the presence of a transparent layer allows the strain pulse to pass through the interface, into the ${\mathrm{Al}}_{2}O_{3}$ layer. That effectively shifts the detection region deeper into the sample and enhances detection sensitivity (Fig. 5(c)) [14].

**Fig. 6:**
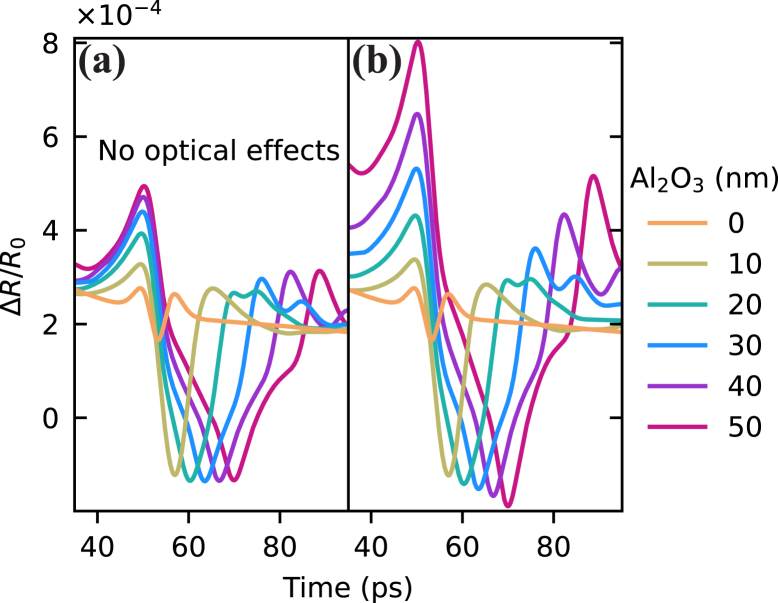
Simulation of the first echo in the freestanding aluminum film excluding (a) and including (b) optical effects for different ${\mathrm{Al}}_{2}O_{3}$ thicknesses. Thermo-optic effects are excluded both in (a) and (b) for clarity. Optical effects are excluded by setting the refractive index of ${\mathrm{Al}}_{2}O_{3}$ for both pump and probe to 1, and strain-optic coefficient of ${\mathrm{Al}}_{2}O_{3}$ to 0.

The signal enhancement due to this free boundary elimination effect increases with top layer thickness, but saturates when the thickness reaches around half the strain pulse extent. In our case this happens at 20–30 nm thickness of ${\mathrm{Al}}_{2}O_{3}$, as shown by simulations (Fig. 6(a)). However, the experiments clearly show that the signal enhancement continues beyond this saturation thickness, which points to the existence of additional effects. By including the influence of optical effects caused by the presence of strain in the ${\mathrm{Al}}_{2}O_{3}$ layer, this continued enhancement can be explained, and good agreement between simulation and experiment can be obtained (Fig. 6). Three main optical effects are identified, namely the strain-optic effect in the ${\mathrm{Al}}_{2}O_{3}$ that changes its refractive index, the physical thickness variation of the ${\mathrm{Al}}_{2}O_{3}$ layer induced by the strain, and the coating effect of the ${\mathrm{Al}}_{2}O_{3}$ layer. The last effect means that for a given strain-induced refractive index change in aluminum, the samples with different thicknesses of ${\mathrm{Al}}_{2}O_{3}$ show different $\Delta R_{0}/R$ signal.

**Fig. 7:**
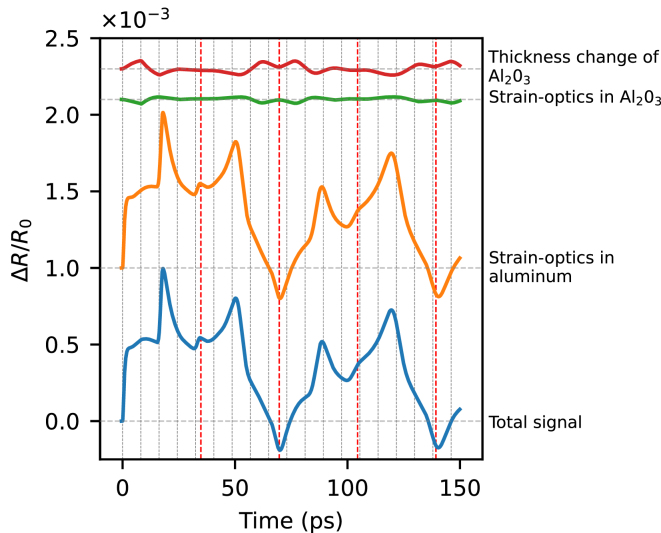
Separated optical effects in aluminum with 50 nm thick ${\mathrm{Al}}_{2}O_{3}$ layer. Vertical black and red lines represent half of the roundtrip time in ${\mathrm{Al}}_{2}O_{3}$ and in the whole sample respectively. Horizontal lines indicate the vertical offset applied for clarity.

To better understand the contribution of these optical effects to the reflectivity curve, it is sufficient to decompose the total signal into individual components. Fig. 7 shows the reflectivity change caused by the thickness change of ${\mathrm{Al}}_{2}O_{3}$ (red curve), by strain-induced refractive index variation of ${\mathrm{Al}}_{2}O_{3}$ (green curve) and by strain-induced refractive index variation of aluminum (orange curve). A linear decomposition of the total signal into these three components is justified as the effects can be treated as a small perturbation of the reflectivity. The roundtrip times in the Al and ${\mathrm{Al}}_{2}O_{3}$ layers are indicated by vertical lines. The green and red curves correspond to strain dynamics in ${\mathrm{Al}}_{2}O_{3}$ and the orange curve to strain dynamics in aluminum. After the pump is absorbed by the aluminum film, two unipolar compressive strain pulses are generated. One pulse propagates into the aluminum and the other into ${\mathrm{Al}}_{2}O_{3}$. The strain pulse in the ${\mathrm{Al}}_{2}O_{3}$ rapidly reflects within the layer, changing its polarity upon each reflection from the air/${\mathrm{Al}}_{2}O_{3}$ interface. The polarity change can be observed in the red and green curves as a decaying oscillation with the period of the ${\mathrm{Al}}_{2}O_{3}$ roundtrip time. Upon each full round trip in the ${\mathrm{Al}}_{2}O_{3}$ layer the strain pulse partially couples into the aluminum, giving rise to spikes in the orange curve in Fig. 7. After 2–3 round trips in ${\mathrm{Al}}_{2}O_{3}$, the strain energy is almost fully transferred to the aluminum. Around 50 ps the strain pulse that was originally propagating in the aluminum film reaches the ${\mathrm{Al}}_{2}O_{3}$/aluminum interface, which can be observed as a spike in the orange curve. After a roundtrip in ${\mathrm{Al}}_{2}O_{3}$ this pulse constructively interferes with the following strain pulse that was propagating in ${\mathrm{Al}}_{2}O_{3}$. That interference results in a dip around 70 ps. For a more intuitive overview of these effects, see the time-lapse animation for the case of a 50 nm ${\mathrm{Al}}_{2}O_{3}$ layer in Supplementary Movie 2.

**Fig. 8:**
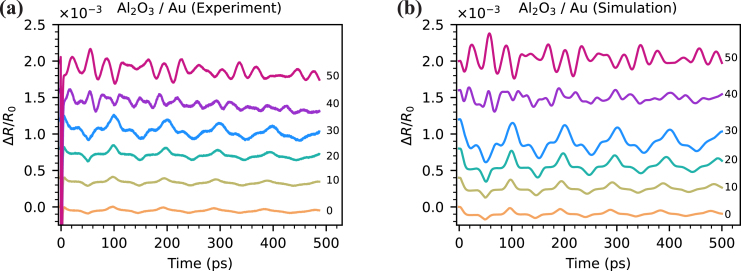
Measured (a) and simulated (b) reflectivity change in ${\mathrm{Al}}_{2}O_{3}$/gold samples. Pump pulse energy 2 nJ. Probe wavelength 390 nm. Gold thickness 160 nm. ${\mathrm{Al}}_{2}O_{3}$ thickness is shown on the graph. A vertical offset between the different traces is applied for clarity. Optical parameters of gold used in simulation: $n_{pump}=0.24+6.7i$, $n_{probe}=1.47+1.94i$, ∂n/∂ɛ=−0.83−1.25i. The strain-optic coefficient is adjusted for better agreement with the experiments. The thermo-optic effect is neglected due to its weak presence in experiments. Optical parameters of ${\mathrm{Al}}_{2}O_{3}$: $n_{pump}=1.67$, $n_{probe}=1.7$, and ∂n/∂ɛ=−0.4.

From Fig. 7 one can see that the total signal is mostly determined by the strain-induced refractive index modulation in aluminum. This is because aluminum has relatively strong strain-optic coupling. For aluminum we use the value ∂n/∂ɛ=−29.19−25.15i which has the same phase as in [36]. The amplitude of ∂n/∂ɛ was adjusted for a better fit with experimental results. For ${\mathrm{Al}}_{2}O_{3}$ we use ∂n/∂ɛ=−0.4 which agrees well with our experimental results. From this analysis, the influence of the thickness change and strain-optic coupling in ${\mathrm{Al}}_{2}O_{3}$ is found to be weak, and the coating effect of the ${\mathrm{Al}}_{2}O_{3}$ layer is identified as the main reason for the optical enhancement of the strain detection. Furthermore, as the thickness change of ${\mathrm{Al}}_{2}O_{3}$ and strain-optic effects in ${\mathrm{Al}}_{2}O_{3}$ counteract each other, the coating effect becomes even more apparent. Such a destructive interference effect is the result of a negative strain-optic coefficient in most materials. While the positive strain expands the ${\mathrm{Al}}_{2}O_{3}$ layer, the average refractive index of the layer is decreased due to the negative strain-optic coefficient. As a result, the contribution of these two effects on the phase accumulation within the ${\mathrm{Al}}_{2}O_{3}$ layer is partially canceled.

### 4.2 Photoacoustic signals in gold films

Photoacoustic signals as observed in ${\mathrm{Al}}_{2}O_{3}$/gold bi-layers are shown in Fig. 8. In these structures, a similar signal enhancement for increasing ${\mathrm{Al}}_{2}O_{3}$ layer thickness is observed, but it is accompanied by much more dramatic changes in the shape of the signals as compared to the ${\mathrm{Al}}_{2}O_{3}$/aluminum bi-layers.

**Fig. 9:**
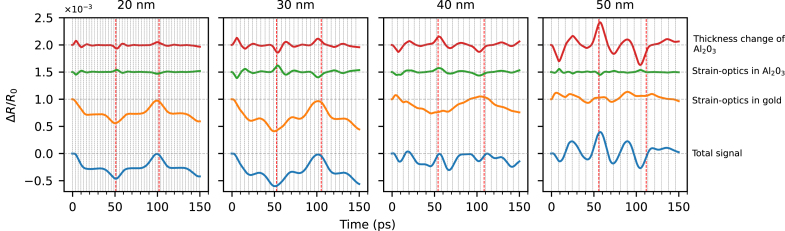
Separated optical effects in gold with different thickness of ${\mathrm{Al}}_{2}O_{3}$ layer. Vertical black and red lines represent half of the roundtrip time in ${\mathrm{Al}}_{2}O_{3}$ and in the whole sample respectively. Horizontal lines indicate the vertical offset applied for clarity.

To begin with, the reflectivity change curve shape for thin layers of ${\mathrm{Al}}_{2}O_{3}$ requires some explanation since it strongly differs from the aluminum case. Gold has much lower electron–phonon coupling and higher electron heat conductivity compared to aluminum. In our simulations we used values $G=2.45\times 10^{16}\;{Wm}^{-3}K^{-1}$ and $k_{e}\left(300\;K\right)=315\;{Wm}^{-1}K^{-1}$ for gold and $G=3\times 10^{17}\;{Wm}^{-3}K^{-1}$ and $k_{e}\left(300\;K\right)=235\;{Wm}^{-1}K^{-1}$ for aluminum [27], [28]. Those differences lead to a broadening of the pump-heated region. Specifically in our case, the gold layer becomes almost homogeneously heated, with expansion starting from both edges. Two expansion fronts then move towards the center of the layer at the speed of sound, forming two step-like strain pulses. The first detected strain pulse at 50 ps is the one that is generated on the back surface of the sample. Because it does not undergo a sign-flip due to reflection, it has an opposite polarity compared to the first echo pulse at 100 ps (see the time-lapse animation in Supplementary Movie 3). Thus, the reflectivity curves for 0–30 nm thick ${\mathrm{Al}}_{2}O_{3}$ (Fig. 8) contain two sequences of signals that are staggered in time, one being positive and the other negative. The echo decay is weaker than in the case of ${\mathrm{Al}}_{2}O_{3}$/aluminum samples due to higher acoustic impedance mismatch between gold and PMMA. For $\nu_{Au}=3350\;m/s$ and $\rho_{Au}=19\;280\;kg/m^{3}$ the reflectivity at the gold-PMMA interface is 90 %. The gold viscosity parameter η is adjusted to a value 2 × 10^-2^ Pa s.

To understand the significant signal shape change that happens for layer thicknesses upward of 40 nm of ${\mathrm{Al}}_{2}O_{3}$ (Fig. 8), we do a signal decomposition (Fig. 9) similar to the ${\mathrm{Al}}_{2}O_{3}$/aluminum case (Fig. 7).

**Fig. 10:**
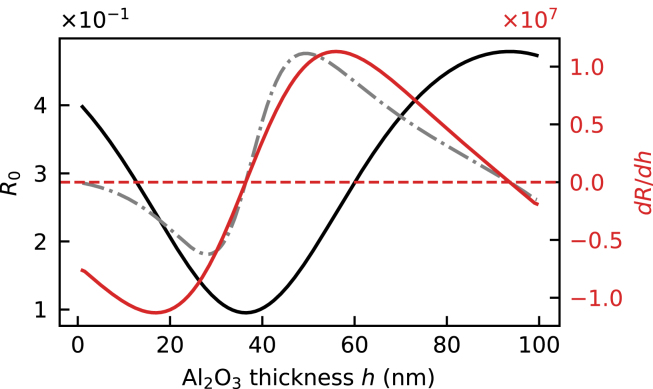
Calculated reflectivity of the ${\mathrm{Al}}_{2}O_{3}$/gold stack (black solid curve) as a function of ${\mathrm{Al}}_{2}O_{3}$ thickness. The red solid curve is a thickness derivative of the reflectivity. The gray dash-dotted curve shows the arbitrarily y-scaled $\frac{\Delta R/R_{0}}{ɛ}$ value ($\frac{dR}{dh}\approx \frac{\Delta R}{hɛ}\to \frac{\Delta R}{hɛ}\cdot \frac{h}{R_{0}}=\frac{\Delta R/R_{0}}{ɛ}$).

The effect of a thickness change of ${\mathrm{Al}}_{2}O_{3}$ (Fig. 9, red trace) has the same behavior as in the ${\mathrm{Al}}_{2}O_{3}$/aluminum stack (Fig. 7). However, a sign flip of the curve is observed at 40 nm of ${\mathrm{Al}}_{2}O_{3}$. The reason for this sign flip is visualized in Fig. 10, which shows the reflectivity of the ${\mathrm{Al}}_{2}O_{3}$/gold stack as a function of ${\mathrm{Al}}_{2}O_{3}$ thickness. Around 35 nm the reflectivity curve has a local minimum that corresponds to the point where the derivative crosses zero, thus changing the sign of the reflectivity change due to layer thickness variation. The reflectivity change is determined both by the slope of the reflectivity curve and by the total deformation, i.e. the integral of strain. While the thickness of the ${\mathrm{Al}}_{2}O_{3}$ layer does not affect the amplitude of the strain pulse, it does influence the deformation of the layer. As long as the ${\mathrm{Al}}_{2}O_{3}$ layer thickness is smaller than the strain pulse length, the total deformation (namely Δh in Fig. 10) increases with ${\mathrm{Al}}_{2}O_{3}$ thickness, causing a higher reflectivity change even if the slope is the same. Furthermore, the echo signals are defined by the $\Delta R/R_{0}$ value (Fig. 8) which depends on the nominal reflectivity $R_{0}$ and therefore also on the ${\mathrm{Al}}_{2}O_{3}$ thickness. Thus, the $\frac{\Delta R/R_{0}}{ɛ}$ value (Fig. 10 dash-dotted gray) gives the best intuition for the amplitude of the signals induced by thickness change of ${\mathrm{Al}}_{2}O_{3}$.

For an ${\mathrm{Al}}_{2}O_{3}$ layer thickness up to 40 nm, the strain-optic effect in ${\mathrm{Al}}_{2}O_{3}$ (Fig. 9, green) behaves as discussed in Paragraph 4.1, showing an opposite effect on ΔR as the thickness changes. At layer thicknesses of 40 nm and higher, this relation changes. At this point, the ${\mathrm{Al}}_{2}O_{3}$ thickness becomes comparable to the probe wavelength. Thus, the non-homogeneous distribution of the strain-induced refractive index change in ${\mathrm{Al}}_{2}O_{3}$ cannot be approximated by an effective average change of refractive index of the ${\mathrm{Al}}_{2}O_{3}$ layer.

A similar evolution happens with the part of the signal that corresponds to the strain-optic coupling in gold (Fig. 9 orange). Although, here it is due to the complexity of the refractive index profile within the penetration depth of the probe light in gold. Multiple strain pulses that evolve in the sample due to partial reflections at the ${\mathrm{Al}}_{2}O_{3}$/gold interface effectively create a dynamic multi-layered stack (see Supplementary Movie 4).

From Fig. 9 one can see that the strain dynamics in gold is the main contribution to the total signal for the 20 nm and 30 nm thick ${\mathrm{Al}}_{2}O_{3}$, while for the 40 nm and 50 nm the total signal is mostly determined by the strain dynamics in ${\mathrm{Al}}_{2}O_{3}$. This change in the dominant mechanism responsible for a ΔR variation explains the significant reflectivity curve shape change that is observed for thicker layers of ${\mathrm{Al}}_{2}O_{3}$ in Fig. 8. The more prominent effect of the strain dynamics in ${\mathrm{Al}}_{2}O_{3}$ for ${\mathrm{Al}}_{2}O_{3}$/gold bi-layers compared to ${\mathrm{Al}}_{2}O_{3}$/aluminum ones in the same range of ${\mathrm{Al}}_{2}O_{3}$ thickness is due to a shorter probe wavelength (390 nm vs 780 nm) and lower strain-optic coefficient of gold (−0.83−1.25j vs −29.19−25.15i).

## 5 Conclusions

The effect of transparent nanolayers on the optical detection of light-induced strain waves has been systematically studied. We showed that, in addition to signal enhancements caused by free boundary effects [14], the presence of a transparent layer introduces several additional optical phenomena that influence the detection sensitivity. These optical effects were classified into three types: the coating effect of the transparent layer, the strain-optic effect in the transparent layer, and a physical thickness variation of the transparent layer induced by the strain. It was shown that the relative contribution of each type of these optical effects to the total $\Delta R/R_{0}$ signal depends on the thickness of the transparent layer. Through the interplay of these different effects, the time-dependent variation in $\Delta R/R_{0}$ can display complex structure compared to a bare metal film. Although it is impossible to observe experimentally all three optical effects separately, theoretical calculations of the total $\Delta R/R_{0}$ show good agreement with experimental results.

## CRediT authorship contribution statement

**Maksym Illienko:** Writing – original draft, Validation, Software, Methodology, Investigation, Formal analysis, Data curation, Conceptualization. **Matthias C. Velsink:** Writing – review & editing, Validation, Software, Methodology, Data curation, Conceptualization. **Stefan Witte:** Writing – review & editing, Validation, Supervision, Resources, Project administration, Methodology, Investigation, Funding acquisition, Conceptualization.

## Declaration of competing interest

The authors declare that they have no known competing financial interests or personal relationships that could have appeared to influence the work reported in this paper.

## Data Availability

Data will be made available on request.

## Appendix A Supplementary data

Video S1 Simulation of the propagation of a light-induced strain pulse in a 171nm aluminum membrane (left) and the resulting strain-induced reflectivity change ΔR/R0 (right).

Video S2 Simulation of the propagation of a light-induced strain pulse in a 171nm aluminum membrane with a 50nm Al${}_{2}$O${}_{3}$ top layer (left), and the resulting strain-induced reflectivity change (right).

Video S3 Simulated propagation of a light-induced strain pulse in a 160nm gold membrane (left) and the strain-induced reflectivity change (right).

Video S4 Simulated propagation of a light-induced strain pulse in a 160nm gold membrane with a 50nm Al${}_{2}$O${}_{3}$ top layer (left), and the strain-induced reflectivity change (right).

Video S5 Simulated propagation of a light-induced strain pulse in a 160nm gold membrane with a 50nm Al2O3 top layer (left), and the strain-induced reflectivity change (right).

MMC S1 Supplementary information about the simulation movies.
