# Supplementary material for: Understanding photoacoustic signal formation in the presence of transparent thin films
Source: Photoacoustics. 2024 May 13;38:100617. doi: 10.1016/j.pacs.2024.100617 (PMC11637020; doi:10.1016/j.pacs.2024.100617)
Supplement: MMC S1 — Supplementary information about the simulation movies. [file mmc6.pdf]

Supplemental Material for

Understanding photoacoustic signal formation in the presence of transparent thin films

Maksym Illienko[1], Matthias C. Velsink[1], and Stefan Witte[*1,2]

[1]*Advanced Research Center for Nanolithography (ARCNL), Science Park 106, Amsterdam, 1098 XG, The Netherlands*
[2]*Department of Physics and Astronomy, Vrije Universiteit, De Boelelaan 1081, Amsterdam, 1081 HV, The Netherlands*

A set of video animations is provided for an intuitive understanding of $\Delta R/R_0$ signal formation in metal films with and without an Al_2O_3 top layer. All the material parameters used to simulate animations are the same as those used for simulations in the main text. The pump and probe are incident on the sample from the left side in all the animations. Metal films are depicted in beige and the Al_2O_3 layer in light blue.

Supplementary Movie1 shows the propagation of a light-induced strain pulse in a $171\,\mathrm{nm}$ aluminum membrane and the strain-induced reflectivity change $\Delta R/R_0$.

Supplementary Movie2 shows the propagation of a light-induced strain pulse in a $171\,\mathrm{nm}$ aluminum membrane with a $50\,\mathrm{nm}$ Al_2O_3 top layer and the strain-induced reflectivity change. The $\Delta R/R_0$ signal is decomposed into three components described in the main text: the thickness change of the Al_2O_3 layer (red), and the strain-induced refractive index variation in the Al_2O_3 layer (green) and aluminum (orange).

Supplementary Movie3 shows the propagation of a light-induced strain pulse in a $160\,\mathrm{nm}$ gold membrane and the strain-induced reflectivity change $\Delta R/R_0$. This animation is very convenient for estimating the detection region in the gold layer. By detection region, we mean the depth within which the strain pulse can be detected by the probe pulse. Two step-like strain pulses are generated after the pump pulse hits the sample. The strain pulse that propagates from the left side of the sample causes a decrease in reflectivity. At the moment the front of this strain pulse leaves the detection region, the $\Delta R/R_0$ curve flattens. In our case this happens around $18\,\mathrm{ps}$ corresponding to a detection region of around $50\,\mathrm{nm}$ (light green). Note that the detection region is significantly longer than a penetration depth conventionally defined as $\lambda/4\pi\kappa$, where λ is the probe wavelength and κ is the imaginary part of the complex refractive index. For the values $\lambda = 390\,\mathrm{nm}$ and $\kappa = 1.94$ used in our simulations the penetration depth is only $16\,\mathrm{nm}$.

Supplementary Movie4 shows the propagation of a light-induced strain pulse in a $160\,\mathrm{nm}$ gold membrane with a $50\,\mathrm{nm}$ Al_2O_3 top layer and the strain-induced reflectivity change $\Delta R/R_0$. The detection region in gold is shown by light green. The $\Delta R/R_0$ signal is decomposed into three components described in the main text: the thickness change of the Al_2O_3 layer (red), and the strain-induced refractive index variation in the Al_2O_3 layer (green) and gold (orange). The strain distribution within the Al_2O_3 layer and the detection region is strongly heterogeneous. Furthermore, both the thickness of Al_2O_3 and the width of the detection region are comparable to the probe wavelength. These two aspects lead to a complicated time dependence of the reflectivity change caused by strain-induced refractive index variation in Al_2O_3 and gold (green and orange curves).

Supplementary Movie5 shows electron and lattice temperature dynamics in $171\,\mathrm{nm}$ aluminum and $160\,\mathrm{nm}$ gold membranes within the first $5\,\mathrm{ps}$ after pump absorption. Due to its higher electron heat conductivity and weaker electron-phonon coupling, the gold film gets nearly homogeneously heated. In contrast, temperature gradients in the aluminium remain more distinct.

[*]s.witte@arcnl.nl
